# Differing contributions of inferior prefrontal and anterior temporal cortex to concrete and abstract conceptual knowledge

**DOI:** 10.1016/j.cortex.2014.09.001

**Published:** 2015-02

**Authors:** Paul Hoffman, Richard J. Binney, Matthew A. Lambon Ralph

**Affiliations:** a Neuroscience and Aphasia Research Unit (NARU), University of Manchester, UK; b Eleanor M. Saffran Center for Cognitive Neuroscience, Temple University, Philadelphia, PA, USA

**Keywords:** Concreteness, Imageability, Semantic cognition, Verbal comprehension, Anterior temporal lobe

## Abstract

Semantic cognition is underpinned by regions involved in representing conceptual knowledge and executive control areas that provide regulation of this information according to current task requirements. Using distortion-corrected fMRI, we investigated the contributions of these two systems to abstract and concrete word comprehension. We contrasted semantic decisions made either with coherent contextual support, which encouraged retrieval of a rich conceptual representation, or with irrelevant contextual information, which instead maximised demands on control processes. Inferior prefrontal cortex was activated more when decisions were made in the presence of irrelevant context, suggesting that this region is crucial for the semantic control functions required to select appropriate aspects of meaning in the face of competing information. It also exhibited greater activation for abstract words, which reflects the fact that abstract words tend to have variable, context-dependent meanings that place higher demands on control processes. In contrast, anterior temporal regions (ATL) were most active when decisions were made with the benefit of a coherent context, suggesting a representational role. There was a graded shift in concreteness effects in this region, with dorsolateral areas particularly active for abstract words and ventromedial areas preferentially activated by concrete words. This supports the idea that concrete concepts are closely associated with visual experience and abstract concepts with auditory-verbal information; and that sub-regions of the ATL display graded specialisation for these two types of knowledge. Between these two extremes, we identified significant activations for both word types in ventrolateral ATL. This area is known to be involved in representing knowledge for concrete concepts; here we established that it is also activated by abstract concepts. These results converge with data from rTMS and neuropsychological investigations in demonstrating that representational content and task demands influence recruitment of different areas in the semantic network.

## 1 Introduction

Storing and processing word meanings involves a widely distributed network of brain regions. Investigating how elements of this network respond to different types of word can provide important insights into the functional organisation of the system. This study focused on differential activations during comprehension of concrete versus abstract words (e.g., *rope vs* *hope*). Two main classes of theory have been proposed to account for these. The first class claims that concrete and abstract words differ in terms of their *representational substrate*. It is often claimed that abstract words have weak or impoverished semantic representations (Jones, 1985; Plaut & Shallice, 1993; Wiemer-Hastings & Xu, 2005). Jones (1985), for example, found that participants judged it easier to predicate (i.e., generate factual statements for) concrete concepts than for abstract. This representational weakness for abstracts might come about because they lack information gained from sensory experience. The most well-known of these is dual-coding theory (Paivio, 1986), which states that while both concrete and abstract concepts are used and experienced verbally, only concrete words are associated with sensory-perceptual information acquired through direct experience of their referents. Paivio proposed that verbal and sensory-perceptual information were represented in separate stores and that concrete words benefited from dual-coding in both stores, while abstract words were represented only in the verbal store. Recent studies have explored other aspects of experience that might be particularly salient for abstract concepts. Abstract words are more strongly associated with emotion and valence responses (Kousta, Vigliocco, Vinson, Andrews, & Del Campo, 2011; Vigliocco et al., 2014), for example and some abstract words are closely linked to spatial and temporal relationships (Troche, Crutch, & Reilly, 2014). These two lines of research indicate that (a) abstract words tend to have less detailed semantic representations than concrete words and (b) the representations of concrete and abstract words are associated with differential involvement of perceptual-motor, verbal and affective neural systems.

An alternative perspective holds that the meanings of abstract words are heavily dependent on the linguistic context in which they are being used (in line with the idea that knowledge of abstract words is tied strongly to language use). Initial evidence for this proposal was presented by Schwanenflugel and colleagues (Schwanenflugel, Harnishfeger, & Stowe, 1988; Schwanenflugel & Shoben, 1983), who noted that when participants were presented with an abstract word, they found it hard to generate a plausible context in which it could be used. More recently, Hoffman, Lambon Ralph, and Rogers (2013) conducted a quantitative analysis of the contextual usage of a large set of words, using a measure of contextual variability called *semantic diversity*. They found that abstract words tended to appear in a broader variety of contexts than did concrete words. We have argued that the greater semantic diversity of abstract words means that they place greater demands on executive *semantic control* processes that provide top-down regulation of knowledge (Hoffman, Jefferies, & Lambon Ralph, 2010; Hoffman, Rogers, & Lambon Ralph, 2011). Semantic control processes interact with semantic representations to ensure that the information accessed at any given moment is appropriate to the current task and context (Badre & Wagner, 2002; Jefferies, 2013; Jefferies and Lambon Ralph, 2006; Thompson-Schill, D'Esposito, Aguirre, & Farah, 1997). Because abstract words can occur in many different contexts, with different semantic information potentially required in each, top-down control of knowledge retrieval is thought to be particularly critical for successful comprehension of these words.

In summary, there are two perspectives on the nature of differences between concrete and abstract words, one proposing differences in the types and quantity of semantic knowledge involved in each and one proposing differential involvement of semantic control processes in each as a result of contextual variability. These two perspectives have often been treated as competing hypotheses (e.g., Binder, Westbury, McKiernan, Possing, & Medler, 2005). In this study, we evaluated a different possibility: namely that both perspectives are correct but that they apply to different neural regions within the semantic network. Semantic control is most strongly associated with the left inferior frontal gyrus (IFG) (Badre & Wagner, 2007; Thompson-Schill et al., 1997). This region shows increases in activation when participants select among semantic competitors (Badre, Poldrack, Pare-Blagoev, Insler, & Wagner, 2005; Thompson-Schill et al., 1997) and when semantic ambiguity must be resolved (Bedny, McGill, & Thompson-Schill, 2008; Rodd, Davis, & Johnsrude, 2005; Zempleni, Renken, Hoeks, Hoogduin, & Stowe, 2007). In addition, lesions to left prefrontal cortex are associated with deficits in semantic control (Metzler, 2001; Noonan, Jefferies, Corbett, & Lambon Ralph, 2010; Robinson, Shallice, Bozzali, & Cipolotti, 2010) and TMS to this region selectively impairs semantic task performance when control demands are high (Whitney, Kirk, O'Sullivan, Lambon Ralph, & Jefferies, 2011, Whitney, Kirk, o'Sullivan, Lambon Ralph, & Jefferies, 2012). The semantic control hypothesis predicts that this area should show increased activation for abstract relative to concrete words (referred to hereafter as an A > C effect) because their variable meanings require greater executive regulation. A > C effects have been reported in IFG (Binder, Desai, Graves, & Conant, 2009; Wang, Conder, Blitzer, & Shinkareva, 2010) but they have not been linked specifically to executive control demands. Other researchers have suggested instead that IFG is involved in representing logical propositions that are key to the meaning of abstract concepts (Shallice & Cooper, 2013) or in integrating or “unifying” semantic knowledge of a word with prior context (Hagoort, 2005). Although most research has focused on the role of left IFG in semantic control, recent studies suggest that other regions, including posterior middle temporal gyrus, are also involved in this function (Noonan et al., 2010; Whitney, Jefferies, & Kircher, 2011; Whitney, Kirk, et al., 2011).

In contrast, the anterior temporal lobes^1^ (ATL) are associated with the representation of semantic knowledge. ATL involvement in multi-modal conceptual knowledge has been observed in studies using H_2_O-PET (Sharp, Scott, & Wise, 2004; Vandenberghe, Price, Wise, Josephs, & Frackowiak, 1996), distortion-corrected fMRI (Binney, Embleton, Jefferies, Parker, & Lambon Ralph, 2010; Visser & Lambon Ralph, 2011), MEG (Marinkovic et al., 2003) and rTMS (Pobric, Jefferies, & Lambon Ralph, 2007; Pobric, Jefferies, & Lambon Ralph, 2010). It is demonstrated most strikingly in the syndrome of semantic dementia, in which atrophy to this area results in selective yet progressive and eventually profound impairment to verbal and non-verbal semantic knowledge (Bozeat, Lambon Ralph, Patterson, Garrard, & Hodges, 2000; Patterson, Nestor, & Rogers, 2007). According to the representational substrates perspective, areas of ATL specialised for representing verbal aspects of knowledge should show an A > C effect while the reverse should be true for areas specialised for representing visual object properties. In other words, the likelihood of observing concreteness effects in the ATL should depend on the degree to which portions of this brain region are specialised for verbal versus visual processing. Some parts of the ATL do show graded specialisation of this sort. The superior ATL shows greater activation for semantic processing of auditory and verbal stimuli, relative to pictures (Moore & Price, 1999; Visser, Jefferies, Embleton, & Lambon Ralph, 2012; Visser & Lambon Ralph, 2011). This specialisation may arise because this area is strongly connected to primary auditory processing regions in posterior STG (Binney, Parker, & Lambon Ralph, 2012). Consistent with the idea that abstract words are especially dependent on verbal processing regions, A > C effects have been observed in this area in previous studies (Binder et al., 2009; Noppeney & Price, 2004; Tettamanti et al., 2008; Wang et al., 2010). Conversely, the ventromedial ATL has strong connections with visual processing regions in ventral posterior temporal cortex (Binney et al., 2012) and shows greater activation when participants make semantic decisions to pictures relative to words (Visser et al., 2012). This visual semantic bias suggests that a C > A effect might be expected in this area, since concrete words are more strongly associated with visual experiences.

It is also important to note that other parts of the ATL are equally responsive to all meaningful stimuli, no matter which modality they are presented in. PET and recent distortion-corrected fMRI studies have identified an area in the inferior temporal and fusiform gyri (which we term here the ventral ATL) that responds equivalently to spoken words, written words, pictures and non-verbal sounds (Spitsyna, Warren, Scott, Turkheimer, & Wise, 2006; Vandenberghe et al., 1996; Visser et al., 2012). Hypometabolism in this region has been linked to multi-modal semantic deficits in patients with semantic dementia (Butler, Brambati, Miller, & Gorno-Tempini, 2009; Mion et al., 2010) and it has been proposed that the ventral ATL acts as a multi-modal convergence “hub” that integrates information from modality-specific sites across the brain to form conceptual representations (Binney et al., 2012; Patterson et al., 2007). While a number of recent neuroimaging studies have demonstrated activation in ventral ATL for concrete concepts (Peelen & Caramazza, 2012; Robson et al., 2014; Visser et al., 2012; Visser & Lambon Ralph, 2011), we are not aware of any studies reporting activation in this area for abstract words. This may be in part due to susceptibility artefacts that make it difficult to obtain reliable signal in this area with standard, gradient-echo fMRI (Devlin et al., 2000; Visser, Jefferies, & Lambon Ralph, 2010). While special steps can be taken in image acquisition and processing to combat this problem (e.g., Embleton, Haroon, Morris, Lambon Ralph, & Parker, 2010; Halai, Welbourne, Embleton, & Parkes, 2014), the vast majority of fMRI studies do not do so and have reduced sensitivity to activation in the ventral ATLs. It is important to address the question of ventral ATL involvement in abstract concepts because, in common with much of the literature on semantic cognition, implemented computational models of the hub theory have focused exclusively on concrete concepts (Lambon Ralph, Lowe, & Rogers, 2007; Rogers et al., 2004). As a consequence, Shallice and Cooper (2013) have recently proposed that a separate system is required to meet the different challenges of representing abstract concepts. Furthermore, some researchers have proposed that ATL atrophy in semantic dementia primarily affects visual feature knowledge and, as a consequence, has a disproportionate effect on understanding of concrete words (Bonner et al., 2009; Libon et al., 2013). This theory predicts minimal activation in the ventral ATL when participants comprehend abstract words (though some neuropsychological evidence contradicts this view; see General Discussion).

To summarise, the semantic control hypothesis predicts an A > C effect in IFG on the basis that comprehending abstract words is executively demanding due to their variable, context-dependent meanings. The representational substrates perspective predicts that both A > C and C > A effects may arise in different subregions of the ATL, due to graded specialisations within superior and ventromedial ATL for verbal versus visual semantic knowledge respectively. The ventral ATL is known to play an important role in the processing of concrete words but its involvement in abstract word knowledge is unclear, with some theories predicting that it is minimally involved. Furthermore, previous studies have not distinguished between effects associated with executive control and those associated with knowledge representation. In this study we used a novel cueing paradigm to make this distinction. We varied the level of contextual support available while participants made semantic decisions to concrete and abstract words (see Table 1). On some trials, a coherent contextual cue was provided immediately prior to the decision. This allowed the participant to activate relevant conceptual knowledge prior to the decision, reducing the requirement for top-down semantic control processes (Noonan et al., 2010). On other trials, the cues contained irrelevant information, which increased executive demands by introducing conflicting conceptual information that had to be ignored. Regions involved in semantic control would therefore activate more strongly under irrelevant cue conditions. In contrast, we expected regions involved in the representation of conceptual knowledge activate to most strongly when a relevant contextual cue was provided, as this would allow participants to retrieve a greater quantity of coherent semantic information to support their decision. Importantly, we used a distortion-corrected fMRI protocol (Embleton et al., 2010), allowing us to assess concreteness effects in ventral ATL for the first time. As noted above, this region is critical for semantic processing but is poorly sampled in most fMRI studies due to susceptibility artefacts and signal drop-out (Devlin et al., 2000).

In addition, and as a secondary aim of the study, we investigated concreteness effects in areas of the default mode network. C > A effects are frequently observed in the angular gyrus and posterior cingulate (Binder et al., 2005; Sabsevitz, Medler, Seidenberg, & Binder, 2005; Wang et al., 2010), areas which typically display deactivations during task-related processing relative to rest (Buckner, Andrews-Hanna, & Schacter, 2008). Binder et al. (2009) have proposed that the posterior cingulate and, in particular, the angular gyrus are key sites for semantic representation and that concrete regions activate these regions strongly because they have more detailed semantic representations. These proposals might seem at odds with the fact that these areas are generally deactivated during task-related processing. However, there are two factors that could potentially resolve the apparent discrepancy. First, although these areas *typically* deactivate when participants perform demanding tasks, this may not be true of semantic tasks specifically. Second, even if they were more active during rest, strong activation of these regions in task-free situations might indicate daydreaming and undirected thoughts that contain rich semantic content (Binder et al., 1999). If this were the case, then we would expect other elements of the semantic network, including the ATL, to also show greater activity at rest. We therefore assessed the relationship between areas showing semantic effects and areas showing positive or negative activity with respect to a resting baseline.

## 2 Method

### 2.1 Participants

Twenty healthy participants took part (11 male, mean age = 25, range = 20–39). Data from one participant was discarded due to image artefacts. All participants were native English speakers with no history of neurological or psychiatric disorders and normal or corrected-to-normal vision. The study was approved by the local ethics board.

### 2.2 Experimental task

Participants completed a synonym judgement task similar to that used in previous neuropsychological (Jefferies, Patterson, Jones, & Lambon Ralph, 2009), transcranial magnetic stimulation (Hoffman et al., 2010; TMS; Pobric et al., 2007) and fMRI (Binney et al., 2010) studies. On each trial, participants were presented with a written probe word with three choices below it (a semantically related target and two unrelated foils). They were asked to select the word that was most similar in meaning to the probe (see Table 1 for examples). Prior to each synonym judgement, participants were presented with a written cue consisting of two short sentences. On half of the trials, the cue ended with the probe word and placed it in a particular meaningful context (contextual cue condition). On the remaining trials, the cue did not contain the probe and was not related in meaning to the subsequent judgement (irrelevant cue condition). Participants were unaware when reading the cue whether it would be helpful for their next decision, meaning that neural differences between the two conditions only occurred in the decision phase. We assumed that reading the cue would activate semantic information related to its content and that this information would be strongly active when the subsequent synonym judgement was presented. On contextual cue trials, the pre-activated semantic information was highly relevant to the judgement, which was likely to have two effects. First, processing of the probe word would benefit from the prior activation of the word's meaning and its context, leading to the retrieval of a richer semantic representation. Retrieval of a greater quantity of semantic knowledge leads to stronger activation in areas associated with semantic representation (Whitney, Jefferies, et al., 2011). Second, demands on semantic control regions involved in selecting task-appropriate knowledge would be minimised, since context-relevant knowledge was already active prior to making the judgement. On irrelevant cue trials, the reverse was true. Any semantic information activated by the cue would compete with the semantic information required for the synonym judgement, increasing demands on semantic control and selection regions. Moreover, the probe word would be processed without the benefit of any contextual framework, leading to impoverished activation of semantic knowledge and reduced activation in areas underpinning semantic representation.

### 2.3 Stimuli

200 synonym judgement trials were generated; 100 featuring concrete words and 100 featuring abstract words. Psycholinguistic properties for the probes and choice words are provided in Table 2. In common with most previous studies, we defined words as concrete or abstract based on ratings of imageability. These were significantly higher for concrete words than for abstract words (*t* = 82, *p* < .001). Concrete and abstract trials were matched for log word frequency. The concrete and abstract probes were equal in word length, though the choice words were slightly longer in the abstract condition. Abstract words were also, on average, lower in concreteness and familiarity than concrete words and were later acquired. We also obtained semantic diversity values for all words, which is a measure of the degree of variation among the different contexts in which a word can be used (Hoffman, Lambon Ralph, et al., 2013). Abstract words had significantly higher semantic diversity values than concrete words, indicating that they tend to appear in a broader range of linguistic contexts.

A contextual cue was created for each trial. The cues were between seven and sixteen words in length and consisted of two sentences that placed the probe word in a particular meaningful context. Each cue ended with the probe word. The length of the cue in concrete versus abstract trials did not differ in terms of words or letters (*t* < 1.6, *p* > .1). To generate irrelevant cues, trials were divided into two matched sets A and B and the cues randomly reassigned within each set. Presentation was counterbalanced such that half of the participants saw the set A trials with contextual cues and set B trials with irrelevant cues, and vice versa for the remaining participants. Participants never saw the same trial or cue more than once. We used latent semantic analysis (Landauer & Dumais, 1997) as a means of assessing the strength of relationships between the cues, probes and choice words (see Supplementary Materials for details). Critically, we found that contextual cues had a stronger semantic relationship with their probes and targets than did irrelevant cues. We also found that the relationships between contextual cues, probes and targets were stronger for concrete words than for abstract words. This reflects the fact that abstract words are used in a broad variety of linguistic contexts and therefore share weaker semantic relationships with one another.

### 2.4 Baseline task

As a baseline, we employed a cognitively-demanding number judgement task, again taken from previous neuropsychological, TMS and fMRI studies. On each trial, participants were presented with a probe number between 1 and 99, along with three numerical choices. They were instructed to select the number closest in value to the probe. Previous studies have found that this task was similar in difficulty (in terms of reaction time) to the most demanding synonym judgements (Hoffman et al., 2010; Pobric, Lambon Ralph, & Jefferies, 2009). Therefore, the baseline task required similar levels of attention and general cognitive effort, but minimal semantic processing. Number judgement trials were also preceded by a sentence cue (see Table 1). Therefore, neural processes involved in reading and comprehending the cues were equivalent across all conditions including the baseline, ensuring that differences would only emerge in the judgement phase.

### 2.5 Procedure

Each trial began with a fixation cross presented in the centre of the screen for 500 msec, which was followed by the cue. Participants were instructed to read the cue carefully and to press a button on the response box when they had finished reading. The cue remained on screen for 5000 msec. The judgement probe and three choices were then presented and participants responded by pressing one of three buttons on a response box held in their right hand. The stimuli remained on screen for 4000 msec, at which point the next trial began. Stimuli were presented in blocks of two trials (total duration = 19 sec) with the two trials in each block being taken from the same experimental condition. There were 150 blocks in total and blocks from different conditions were presented in a pseudo-random order. A fixation block of 19 sec, in which no stimuli were presented, occurred after every five blocks of task. We used a blocked design to maximise power; however, this did introduce a degree of predictability in the order of contextual versus irrelevant cues. This is important as it could influence participants' processing of the cues. If a participant became aware that irrelevant cued trials occurred in pairs, they might process the cue less fully on the second trial of the pair. In reality, this is less of a problem than one might expect, for the following reasons. First, blocks followed one another continuously, making it hard to detect when a new block was starting. Second, sometimes two blocks of the same cue type were presented consecutively, making it harder for participants to recognise the blocked structure.

### 2.6 Imaging procedure

A key aim of the study was to assess concreteness effects in the ventral anterior temporal lobe (vATL). Imaging this area with conventional gradient-echo fMRI is affected by magnetic susceptibility artefacts and other technical limitations that result in signal drop-out and distortion (Devlin et al., 2000; Visser et al., 2010). To combat this problem, we employed a spin-echo imaging sequence combined with a post-acquisition distortion-correction, which greatly improves signal quality in the vATL (Embleton et al., 2010). Previous studies have observed robust vATL activations for semantic tasks using this technique (Binney et al., 2010; Visser & Lambon Ralph, 2011). Images were acquired on a 3T Philips Achieva scanner using an 8 element SENSE head coil with a sense factor of 2.5. The spin-echo EPI sequence included 31 slices covering the whole brain with echo time (TE) = 70 msec, time to repetition (TR) = 3200 msec, flip angle = 90°, 96 × 96 matrix, reconstructed in-plane resolution 2.5 × 2.5 mm, slice thickness 4.0 mm 896 images were acquired in total, collected in two runs of 24 min each. Following the standard method for distortion-corrected spin-echo fMRI (Embleton et al., 2010), the images were acquired with a single direction *k* space traversal and a left-right phase encoding direction. In between the two functional runs, a brief “pre-scan” was acquired, consisting of 10 volumes of dual direction *k* space traversal SE EPI scans. This gave 10 pairs of images matching the functional time series but with distortions in both phase encoding directions (10 left-right and 10 right-left). These scans were used in the distortion correction procedure. In addition, a high resolution T1-weighted 3D turbo field echo inversion recovery image was acquired (TR = 8400 msec, TE = 3.9 msec, flip angle 8°, 256 × 205 matrix reconstructed to 256 × 256, reconstructed resolution .938 × .938 mm, and slice thickness of 0.9 mm, SENSE factor = 2.5) with 160 slices covering the whole brain. This image was used for spatial normalisation.

### 2.7 Distortion correction

The spatial remapping correction was computed using the method reported by Embleton et al. (2010). In the first step, each image from the main functional time-series was registered to the mean of the pre-scan images using a 6-parameter rigid-body transformation in SPM8. Subsequently, a spatial transformation matrix was calculated from the pre-scan images, consisting of the spatial re-mapping necessary to correct the distortion. This transformation was then applied to each of the 896 co-registered functional images.

### 2.8 fMRI data analysis

**Fig. 1:**
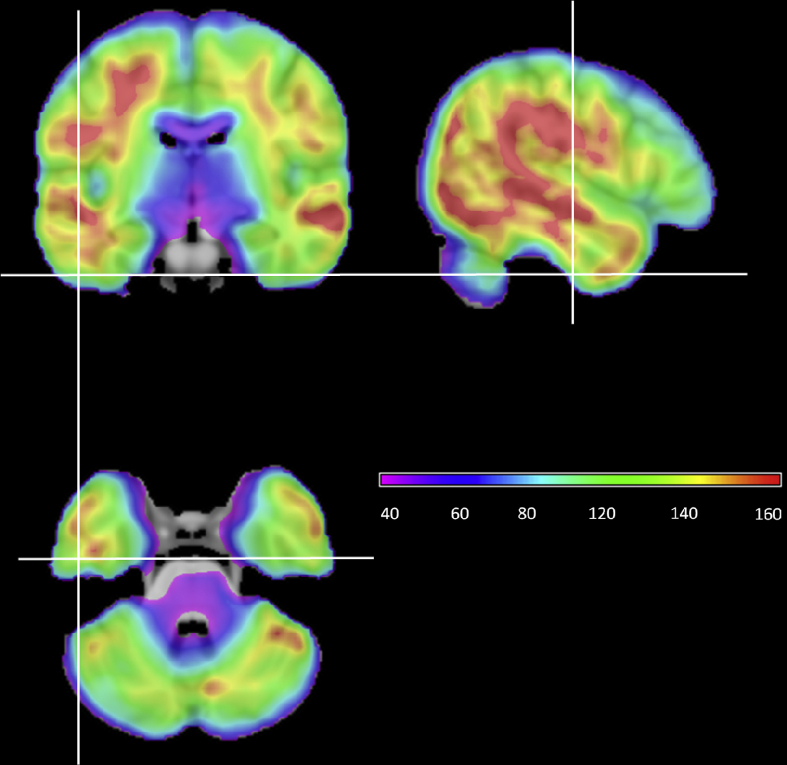
Mean temporal signal-to-noise map. Signal-to-noise was calculated in each participant by dividing the mean intensity in each voxel by its standard deviation. The mean map was obtained by averaging the maps from all participants.

Analysis was carried out using SPM8. The motion and distortion-corrected images for each participant were first co-registered to their T1 structural scan. Spatial normalisation of the T1 scans into MNI space was computed using DARTEL (Ashburner, 2007) and the resulting transformation applied to the functional images, which were resampled to 2 × 2 × 2 mm voxel size and smoothed with an 8 mm FWHM Gaussian kernel. At this point, temporal signal-to-noise (TSNR) maps were generated for each participant by dividing the mean signal in each voxel by its standard deviation (Murphy, Bodurka, & Bandettini, 2007). The mean TSNR map across all participants is shown in Fig. 1. TSNR exceeded 80 in ventral temporal regions. Unlike gradient-echo fMRI, which shows a pronounced drop in TSNR in ventral temporal regions relative to the rest of the brain, TSNR in the ventral temporal lobes was comparable to that in frontal and superior temporal regions.

The data were treated with a high-pass filter with a cut-off of 190 sec and analysed using a general linear model. At the first level, each of the five stimulus conditions was modelled with a separate regressor (concrete-context, concrete-irrelevant, abstract-context, abstract-irrelevant and number baseline). Blocks were modelled with a boxcar function convolved with the canonical haemodynamic response function. Motion parameters were entered into the model as covariates of no interest. Parameter estimates were subjected to several analyses, each targeted at a specific hypothesis.

### 2.9 Concreteness and cueing effects in the frontotemporal semantic network

Our main hypotheses related to condition effects in IFG and ATL regions. We predicted that these areas would show divergent effects with respect to the cueing manipulation and would also show concreteness effects. To identify activated areas in which to test these hypotheses, we first conducted a whole-brain analysis to identify the network involved in making synonym judgements. A contrast was computed for each subject for all semantic conditions combined minus the number baseline and these were submitted to a second-level random effects analysis. A voxel-height threshold of *p* < .001 was adopted for whole-brain analyses. To control for multiple comparisons, a minimum cluster size was determined using a Monte Carlo analysis (Slotnick, Moo, Segal, & Hart, 2003). This modelled the entire image volume, smoothed with a Gaussian kernel of 11 mm FWHM, assumed an individual voxel type-1 error of .001 and ran 1000 simulations to determine the minimum cluster size associated with a corrected *p* < .05. The cluster threshold obtained using this method was 50 voxels. The whole-brain analysis was used to identify regions of interest within the prefrontal and anterior temporal cortices. Concreteness and cue type effects were assessed within ROIs consisting of spheres of 5 mm radius, centred on activation peaks in left IFG, superior ATL (sATL) and ventral ATL (vATL). The Marsbar toolbox (Brett, Anton, Valabregue, & Poline, 2002) was used to obtain contrast estimates in each ROI for each of the semantic conditions relative to the number baseline. Condition effects in each ROI and between ROIs were assessed using ANOVA.

### 2.10 Variation in concreteness effects within the ATL

**Fig. 2:**
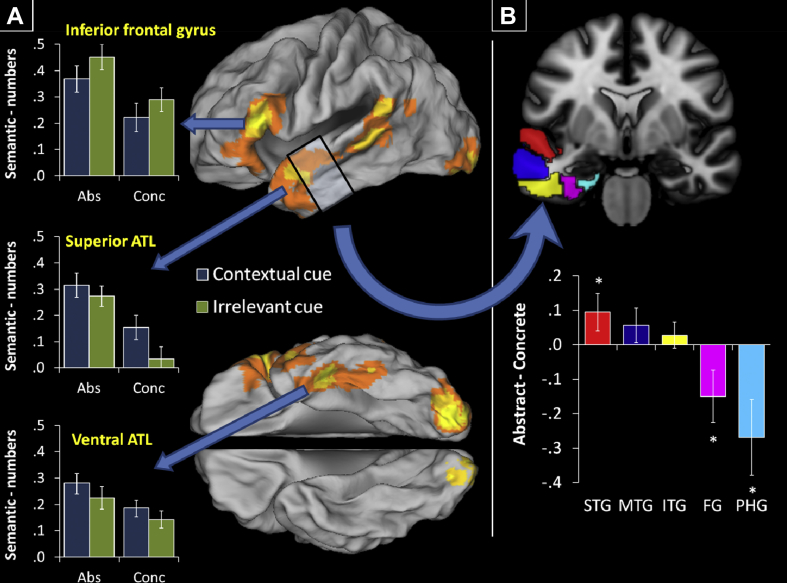
Activations for semantic minus numbers contrast and concreteness effects in the temporal lobe. (A) Activations for semantics – numbers are rendered on the cortical surface (at voxel *p* < .001, cluster size > 50 voxels). Contrast estimates for individual semantic conditions (relative to numbers) are shown for regions of interest in prefrontal and anterior temporal cortex. (B) Contrast estimates for the concreteness effect (i.e., abstract minus concrete) in anterior sections of each temporal gyrus. STG = superior temporal gyrus; MTG = middle temporal gyrus; ITG = inferior temporal gyrus; FG = fusiform gyrus; PHG = parahippocampal gyrus.

As outlined in the Introduction, we predicted that concreteness effects would vary within the ATL as a function of graded specialisations for verbal versus visual semantic knowledge. To test this prediction, we constructed an ROI for each temporal gyrus, based on templates given in the Wake Forest University Pickatlas toolbox (Maldjian, Laurienti, Kraft, & Burdette, 2003). Each gyrus was divided into a number of sections by cutting it in planes perpendicular to the long axis of the temporal lobe. ROI analyses were performed on an anterior section of each gyrus that spanned 20 mm in the *y*-axis, ranging from *y* ≈ −30 to *y* ≈ −10 along the ventral surface (see Fig. 2; we selected this section of the temporal lobe because it displayed the most robust semantic activations, though similar results were obtained in other parts of the lobe). Marsbar was used to extract estimates in each gyrus for the contrast of abstract versus concrete words.

### 2.11 Concreteness effects in other regions

Analyses to this point were targeted on specific frontal and temporal areas. To allow comparison with previous studies, we performed an additional whole-brain analysis comparing concrete with abstract words. We also compared the pattern of concreteness effects with areas of task-related activation and deactivation (i.e., the contrast of the semantic conditions *vs* fixation). Previous studies have reliably identified the angular gyrus and posterior cingulate as showing a C > A activation pattern (Binder et al., 2005; Sabsevitz et al., 2005; Wang et al., 2010). These areas are associated with the default mode network that typically deactivates during stimulus-driven processing (Buckner et al., 2008), as are anterior temporal regions, raising the possibility effects in these areas may relate to differential deactivation rather than task-related increases in activity. To explore this possibility, ROI analyses were conducted for key regions identified in the C > A contrast, based on 5 mm spheres centred on peak co-ordinates.

## 3 Results

### 3.1 Behavioural data

Mean error rates and reaction times in each condition are shown in Table 3. Performance on the number baseline task was comparable to that of the more difficult semantic conditions, confirming that the number task was a suitable baseline for controlling for effects of working memory and attention associated with general cognitive processing. Reaction time data for the semantic task were analysed using a 2 × 2 repeated-measures ANOVA. This revealed main effects of concreteness [*F*(1,18) = 237, *p* < .001] and cue type [*F*(1,18) = 155, *p* < .001]. Abstract words were processed more slowly than concrete words and participants were slower when the judgement was preceded by an irrelevant, rather than contextually appropriate cue. There was also a significant interaction between concreteness and cue type [*F*(1,18) = 25.7, *p* < .001], indicating that the presence of contextual cues benefited abstract words to a greater degree than concrete words. Analysis of error rates replicated these effects [concreteness: *F*(1,18) = 66, *p* < .001; cue type: *F*(1,18) = 45, *p* < .001; interaction: *F*(1,18) = 25.1, *p* < .001]. These effects confirm that the presence of contextual cues aided semantic decisions, presumably by reducing the need for semantic control, and that this benefit was most pronounced for abstract words, which tend to have more variable, context-dependent meanings.

### 3.2 Concreteness and cueing effects in the frontotemporal semantic network

The whole-brain analysis of semantics > numbers revealed a number of peaks in left-hemisphere frontal and temporal regions associated with semantic processing (see Fig. 2; MNI co-ordinates are reported in Table 4). A large IFG cluster was observed, the peak of which fell in pars triangularis (BA45) though it also spanned pars orbitalis (BA47) and encroached onto the most anterior portion of pars opercularis (BA44). Activation was observed along the superior temporal sulcus, running from the ATL into the posterior temporal lobe and extending upward into the supramarginal gyrus (BA40). In line with previous distortion-corrected fMRI studies, robust ventral temporal activation was also found in the fusiform and inferior temporal gyri. This began at *y* ≈ −45 and extended into the ATL, with a peak at *y* = −14 and an anterior extent of *y* ≈ 0. Bilateral occipital activation was also observed, reflecting the greater visual complexity of words relative to numbers.

The effects of the stimulus manipulations in the IFG (pars triangularis peak), superior ATL (sATL) and ventral ATL (vATL) are displayed in Fig. 2A. The data from the these three ROIs were first analysed with a 2 × 2 × 3 repeated-measures ANOVA that included concreteness, cue type and region as factors. This analysis revealed interactions between region and concreteness [*F*(2,36) = 4.52, *p* = .018] and region and cue type [*F*(2,36) = 8.51, *p* = .001]. This indicated that the effects of our experimental manipulations varied across regions; we therefore performed a series of follow-up tests, controlling for multiple comparisons using the false discovery rate (Benjamini & Hochberg, 1995). We first subjected each region to a 2 (concreteness) × 2 (cue type) ANOVA, the results are reported in Table 5. All three regions displayed stronger activation to abstract words but the regions diverged in their response to cueing. IFG showed greater activation when judgements were made following irrelevant cues, consistent with a role in semantic control and selection. In contrast, sATL showed the reverse pattern, with significantly greater activation for judgements made following contextual cues. vATL showed an effect in the same direction as sATL but it was not significant in this region. To determine whether effects differed significantly between pairs of regions, we conducted three pairwise comparisons using 2 × 2 × 2 ANOVAs. These confirmed that the effect of cue type in IFG was significantly different to that in each of the temporal regions [*F*(1,18) > 9.21, *p* < .007], indicating a dissociation in function. In addition, the A > C effect was significantly weaker in the vATL relative to the sATL [*F*(1,18) = 11.6, *p* = .003]. This within-temporal change in the concreteness effect was explored further in the next section.

### 3.3 Variation in concreteness effects within the temporal lobe

We assessed concreteness effects in an anterior section of each temporal gyrus, to test the prediction that there would be a graded shift in responses, with dorsolateral regions showing preferential activation for abstract words and ventromedial regions for concrete words. Fig. 2B shows the results. A one-way ANOVA confirmed that the effect of concreteness varied across the five gyri [*F*(4,72) = 6.25, *p* < .001]. The superior temporal gyrus displayed the strongest preference for abstract words. Middle and inferior gyri displayed smaller (non-significant) A > C effects, while the fusiform and parahippocampal gyri were more strongly activated by concrete words. These patterns are interpreted further in the Discussion.

### 3.4 Concreteness effects in other regions

**Fig. 3:**
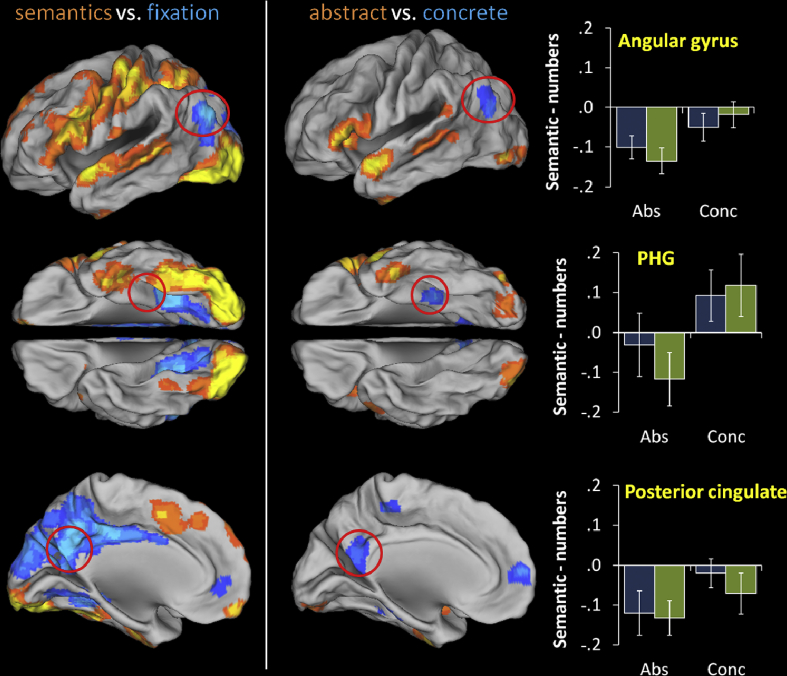
Comparison of concreteness effects with task-related activations and deactivations. Whole-brain analyses of concrete versus abstract decisions and semantic decisions versus fixation (presented at voxel *p* < .001, cluster size > 50 voxels). Contrast estimates for individual semantic conditions (relative to numbers) are shown for areas exhibiting C > A effects.

Results of the whole-brain analysis are displayed in Fig. 3, alongside the analysis contrasting the semantic conditions with fixation. Peak co-ordinates for the concrete versus abstract comparison are reported in Table 6. The A > C contrast identified very similar regions to the overall semantic analysis, including left IFG, ATL, posterior MTG and supramarginal gyrus. This suggests that abstract words place greater demands on the general semantic network, which is reflected behaviourally in slower reaction times for these words. These areas were also positively activated by semantic processing relative to fixation, as seen as in Fig. 3. The C > A contrast identified a number of regions that were outside the network identified by semantics > numbers. Here, we focus on three areas reliably identified in previous studies of concreteness effects (Wang et al., 2010): the mid-parahippocampal gyrus (PHG), the angular gyrus and the posterior cingulate. All of these regions displayed significant C > A effects, which might suggest in role in semantic processing for concrete words. However, both the angular gyrus and posterior cingulate regions also showed overall *deactivation* during semantic processing, relative to fixation (see Fig. 3). The PHG effect fell close to, but did not overlap with, an area of deactivation. These patterns were replicated in ROI analyses that contrasted each semantic condition with the number judgement task. The angular gyrus and posterior cingulate were deactivated in all four semantic conditions, relative to the number judgements. These effects are in stark contrast to temporal and prefrontal cortices, which showed robust positive activations relative to the baseline task and to fixation. PHG was deactivated during abstract word processing but displayed positive activation to concrete words.

## 4 Discussion

Processing differences between concrete and abstract words have long been a source of debate, with one prominent theory arguing either that they differ principally in the types of information involved in representing their meanings, and another that they differ because abstract words have greater contextual variability. We investigated the neural basis of these two theories by investigating differential activations during semantic judgements for concrete and abstract words while also manipulating the degree of contextual support available to guide decisions. Importantly, by utilising distortion-corrected fMRI, we were able to probe all parts of the semantic network, including the ventral ATL, for the first time. We observed the following:

1. Left IFG and superior and ventral ATL areas were amongst those activated by the semantic task. All showed greater activation for abstract words relative to concrete (an A > C effect). However, the response profiles of these areas differed with respect to context. ATL regions showed greater activation when words were processed with the aid of meaningful, coherent contexts. IFG displayed maximal response when cues were irrelevant to the semantic decision.
2. Within the left ATL, we observed a graded shift in concreteness effects moving coronally from dorsolateral to ventromedial cortex. The STG responded most strongly to abstract words while the fusiform and parahippocampal gyri were preferentially activated by concrete words. Middle and inferior temporal gyri lie between these two extremes and showed similar activation for both word types.
3. Outside of the main semantic network, C > A effects were observed in angular gyrus and posterior cingulate. However, these regions were *deactivated* for concrete and abstract words overall, relative to fixation and to the baseline number decisions.

We will discuss these effects in turn.

### 4.1 IFG

It is well-established that the left IFG is involved in the retrieval, selection and regulation of semantic knowledge according to task demands; processes that we refer to here as “semantic control” (Badre & Wagner, 2007; Jefferies and Lambon Ralph, 2006; Thompson-Schill, 2003). In the present study, IFG was robustly activated in all four semantic conditions but showed greater activation for abstract words and when judgements were made following irrelevant cue information. These findings support the role of IFG in semantic control. The irrelevant cue condition required more semantic control for two reasons. First, in the absence of context, a number of possible interpretations and semantic associations for the word may come to mind, requiring semantic control to select the appropriate elements. For example, when processing the word *rate*, participants might initially activate aspects of its meaning associated with prices and costs, which are not relevant to the judgement (the correct synonym was *speed*). In contrast, when judgements are preceded by a congruent context, the appropriate elements of meaning are primed by the cue (e.g., the contextual cue for *rate* was “The new tram is efficient. It moves at a fast rate.”). Second, any semantic information accessed during the reading of the irrelevant cue must be inhibited to prevent it from the influencing the judgement. IFG is involved in inhibiting irrelevant verbal information in a range of tasks (Badre & Wagner, 2005; Thompson-Schill et al., 2002).

**Fig. 4:**
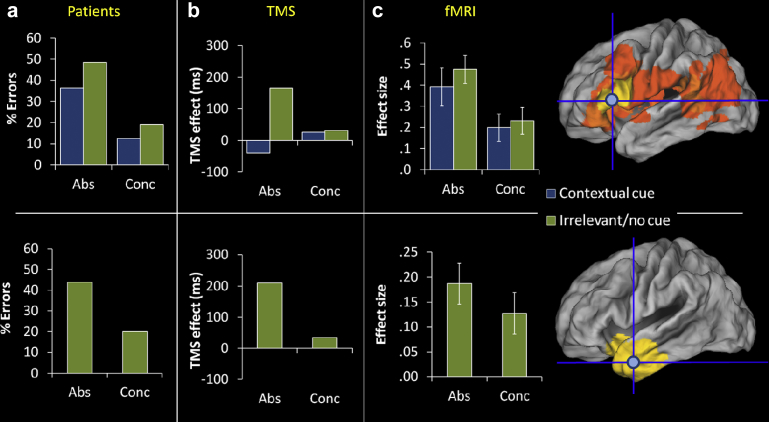
Comparison of fMRI data with previous neuropsychological and TMS studies. fMRI data are presented alongside TMS and patient data originally reported by Pobric et al. (2009), Hoffman et al. (2010) and Hoffman and Lambon Ralph (2011). Top row: (a) error rates for synonym judgements in six patients with lesions to inferior prefrontal cortex, (b) slowing in reaction times following TMS to the same area in healthy subjects, (c) fMRI contrast estimates taken from the co-ordinates stimulated in the TMS study. The brain image shows the lesion overlap map for the patient group and the stimulation site for TMS. Bottom row: (a) error rates for synonym judgements in seven patients with anterior temporal atrophy associated with semantic dementia (b) slowing in reaction times following TMS to the anterior middle temporal gyrus in healthy subjects, (c) fMRI contrast estimates taken from the co-ordinates stimulated in the TMS study. The brain image shows the typical area of hypometabolism in semantic dementia (Nestor, Fryer, & Hodges, 2006) and the stimulation site for TMS.

These findings provide support for the idea that IFG responds more strongly to abstract words because their meanings are inherently more variable than those of concrete words and consequently require more regulation (Bedny & Thompson-Schill, 2006; Hoffman et al., 2010; Hoffman et al., 2011). Schwanenflugel and colleagues (Schwanenflugel et al., 1988; Schwanenflugel & Shoben, 1983) first demonstrated that people find it hard to generate contextual information for abstract words, which was assumed to be because abstract words can occur in many different contexts with associated variations in meaning. This assertion has recently been verified empirically using an objective measure of contextual variability called semantic diversity (Hoffman, Lambon Ralph, et al., 2013). Abstract words tend to appear in a wider range of contexts and, as a consequence, are likely to have more complex and variable meanings. Similarly, studies that have compared words with single versus multiple possible meanings (e.g., *bard vs* *bark*) have reliably found IFG activation for more ambiguous words (Rodd et al., 2005; Zempleni et al., 2007). The present results demonstrate that the need for control in semantic processing is influenced both by the intrinsic semantic variability of the word being comprehended (abstract words being more variable) and by the degree to which word meaning is constrained by preceding context. The association between IFG and semantic control is supported by TMS studies (Whitney, Kirk, et al., 2011, Whitney et al., 2012) and investigations of patients with IFG lesions (Bedny, Hulbert, & Thompson-Schill, 2007; Noonan et al., 2010; Robinson et al., 2010; Thompson-Schill et al., 1998). Fig. 4 presents a direct comparison of the present fMRI results with a previous study that used the same experimental task to explore IFG function in patients with IFG lesions and in healthy participants who received rTMS to the same area (Hoffman et al., 2010). The results from the three methodologies are largely consistent: disruption, either transient or permanent, to the IFG had a more severe effect on abstract words and on trials when contextual information was not available. However, in the previous study there was an interaction between the two factors, which was not significant in the present fMRI data.

The relationship of these findings with Hagoort et al.'s unification hypothesis (Hagoort, 2005; Hagoort, Baggio, & Willems, 2009) is unclear. According to this theory, IFG involvement in semantic processing is due to unification processes that are required to integrate semantic information for individual words into a coherent sentence-level representation. As such, this process should be important for words in the coherent context condition, in which integration of the cue with the subsequent decision probe aids the decision process. What about the irrelevant context condition? One view would be that unification is unlikely to play an important role here, since participants would quickly realise that the cue could not be meaningfully unified with words in the decision trial and to continue to attempt to do so would hamper processing. If this interpretation is correct then one would expect greater IFG activation in the congruent than incongruent condition – which is the opposite pattern to that observed in this study. On the other hand, Hagoort and colleagues have argued that IFG activation indexes the effort involved in attempting to integrate the words into a coherent representation. If participants were engaging in prolonged efforts to integrate the irrelevant cueing information with the words in the decision trial, then this would be compatible with the idea that IFG is involved in semantic unification. A related idea is that IFG is involved in the detection of semantic violations (e.g., Zhu et al., 2012) and that this may account for greater activation in the irrelevant cue condition. This function would be consistent with the more general role of this region in executive regulation of the semantic system.

In considering these findings, it is also important to note that in the contextual cue condition, the probe word for the decision was presented twice during the trial: once in the cue and immediately after during the decision. Our conjecture is that the prior processing of the probe word in a helpful semantic context primed appropriate aspects of knowledge required for the semantic judgement, reducing executive demands. The reduction of IFG activation in this condition is reminiscent of adaptation effects frequently observed in IFG when the same stimuli are repeatedly presented in semantic tasks (Raichle et al., 1994; Thompson-Schill, D'Esposito, & Kan, 1999). Such results have typically been interpreted as indicating reduced need for executive regulation when the relevant semantic information has already been retrieved previously (Fletcher, Shallice, & Dolan, 2000; Thompson-Schill et al., 1999). Importantly, these adaptation effects have been linked specifically to semantic processes and not lower-level perception. Wagner, Koutstaal, Maril, Schacter, and Buckner (2000) demonstrated that adaptation occurred in this region of IFG for words that had been previously encountered in a semantic task but did not when the words had been presented previously in a perceptual judgement task. The reduction in IFG activation, when stimuli are repeated, is therefore consistent with a reduction in semantic control demands when the word has already been processed in a semantically congruent context.

### 4.2 ATL

We found activation in ventral and superior ATL during semantic judgements, which was greater for abstract words. This is consistent with the effects of TMS to this region (Pobric et al., 2009) and the effects of ATL damage in patients with the neurodegenerative syndrome of semantic dementia (Gorno-Tempini et al., 2011; Hoffman and Lambon Ralph, 2011; Jefferies et al., 2009). Fig. 4 demonstrates the close correspondence between the present results and previous TMS and neuropsychological data. The higher spatial resolution of this distortion-corrected fMRI study allowed us to identify separate activation foci in superior and ventral ATL, with distinct response profiles. A > C activation was observed in sATL as in previous studies of concreteness effects (Binder et al., 2009; Wang et al., 2010). However, our novel cueing manipulation indicates that the effect in this area has a different basis to that observed in the IFG. While IFG responded maximally under irrelevant cue conditions, sATL activation was greatest when meanings were processed in a coherent context. This suggests, rather than being involved in executive regulation, sATL may play a role in integrating or enriching a word's meaning based on prior context. sATL is strongly associated with auditory-verbal semantic processing: it shows activation for written and spoken words but not for perceptually-matched non-meaningful stimuli (Scott, Blank, Rosen, & Wise, 2000; Spitsyna et al., 2006). It has also been linked with combinatorial semantic processing (i.e., the extraction of a global meaning from a series of words; Hickok & Poeppel, 2007) and with syntactic aspects of sentence comprehension (Humphries, Binder, Medler, & Liebenthal, 2006) because it activates more strongly to sentence stimuli than to lists of unrelated words (Vandenberghe, Nobre, & Price, 2002). This area was more strongly activated when judgements were made following congruent contextual cues, suggesting that it may be involved in integrating relevant contextual information with the current semantic judgement. This is consistent with involvement combinatorial semantic processing and with the more general role of sATL in verbal comprehension, since discourse processing requires the ongoing integration of information as a conversation unfolds. On this view, sATL showed less activation when the cue was irrelevant because participants rapidly recognised that it was not helpful and disengaged attempts to integrate it. This reduction in activation for irrelevant cues is in direct contrast to IFG and suggests a division of labour, whereby sATL is maximally involved in congruent, contextually enriched language processing while the IFG contribution is greatest under conditions of ambiguity.

The second cluster was in vATL and formed part of a long ribbon of activation running along the border of the fusiform and inferior temporal gyri. fMRI in this area can be affected by susceptibility artefacts and signal drop-out (Devlin et al., 2000; Visser et al., 2010); however, when these technical limitations are addressed it has been found to be robustly activated for concrete concepts in a range of semantic tasks (Binney et al., 2010; Vandenberghe et al., 1996; Visser et al., 2012). Here, we established that this area plays an important role in the representing the meanings of abstract as well as concrete concepts. vATL displayed a similar response across all four semantic conditions. It did show an A > C effect, though this was significantly smaller than that observed in sATL, and it showed no significant difference between the two types of cue. Similarly, in previous studies this region has been found to respond uniformly to semantic judgements for spoken words, written words, pictures and non-verbal sounds (Marinkovic et al., 2003; Spitsyna et al., 2006; Visser & Lambon Ralph, 2011), consistent with that view that the wider ATL region acts as transmodal hub that fuses visual, auditory and other sources of information to form coherent concepts (Lambon Ralph, Sage, Jones, & Mayberry, 2010; Patterson et al., 2007). The role of the ATL hub in representing abstract concepts is less clear and some authors have questioned whether the hub is involved in representing these concepts (Bonner et al., 2009; Meteyard, Cuadrado, Bahrami, & Vigliocco, 2012; Shallice & Cooper, 2013). This view is motivated in part by a number of prominent single-case studies of patients with ATL damage who display a reversal of the typical concreteness effect – i.e., their comprehension of concrete concepts is disproportionately impaired relative to abstract (e.g., Macoir, 2009; Sirigu, Duhamel, & Poncet, 1991; Warrington, 1975). Some recent studies have failed to find this effect in larger case-series of semantic dementia (Hoffman, Jones, & Lambon Ralph, 2013; Hoffman and Lambon Ralph, 2011; Jefferies et al., 2009), suggesting that the “reversal” cases are unusual anomalies, though other studies are inconsistent with this view (Bonner et al., 2009; Loiselle et al., 2012; Yi, Moore, & Grossman, 2007). This apparent variability among patients with ATL damage may be a consequence of variations in the location and extent of damage in different patients. The present study allows for a greater degree of anatomical precision than is possible in neuropsychological studies. We found that a key region of vATL cortex – an area that is strongly linked to semantic deficits in semantic dementia (Mion et al., 2010) – is involved in the processing of abstract words as well as concrete. This suggests that a common temporal lobe system supports comprehension of both word types.

Though the ATL was clearly involved in processing both concrete and abstract words, we also observed graded specialisation in its function. We have recently suggested that there is a degree of graded specialisation within the ATL whereby, due to their differential connections with posterior sensory cortices, conceptual knowledge in the dorsolateral ATL is primarily influenced by auditory-verbal experience and ventromedial ATL by visual information (Binney et al., 2012). Ventrolateral regions lying between these extremes are thought by equally influenced by both. A recent fMRI study supports this view, indicating that pictures activated the anterior fusiform more strongly than words, while the reverse was true in anterior STG (Visser et al., 2012). Here, we have demonstrated for the first time that this graded specialisation can be observed when the conceptual properties of the stimuli are manipulated, rather than their perceptual modality. In the present study, the perceptual input was equivalent for concrete and abstract concepts, since all were written words; however, we observed a graded shift in the ATL corresponding to the conceptual information relevant to each word type. The meanings of abstract words are thought to be specified primarily by their use in language and, accordingly, we observed strong A > C effects in the anterior STS/STG. Conversely, concrete words are additionally associated with visual-perceptual qualities, giving rise to C > A effects in the fusiform and PHG. Inferior temporal gyrus, the site of the vATL peak, showed no significant difference between word types, in line with the equi-modal role established for this area in previous studies (Spitsyna et al., 2006; Vandenberghe et al., 1996; Visser et al., 2012). The most parsimonious explanation for these findings are that the wider ATL region acts as a graded representational space (Binney et al., 2012; Plaut, 2002). According to this computational framework, neurons at the edges of the space display relative specialisation based on the particular sensory inputs they receive while neurons in the centre of the space are equally influenced by all modalities of input. This framework could account for the strong concreteness effects observed at the “edges” of the temporal lobe (i.e., STG and PHG) in terms of their relative specialisations for verbal versus visual inputs, while predicting equi-modal activations in the centre (ITG and the vATL). Importantly, this framework assumes graded specialisation within a single functional system in the ATL, rather than an absolute division into separate processing modules.

### 4.3 Beyond IFG and ATL

Finally, we consider C > A activations observed in other areas of the brain. As in previous studies and meta-analyses, we found C > A effects in angular gyrus, posterior cingulate and mid-PHG. As discussed in the previous section, the activation of PHG most likely reflects retrieval of stored visual characteristics of concepts, which is only possible for highly imageable, concrete words (D'Esposito et al., 1997; Sabsevitz et al., 2005). C > A effects in the angular gyrus and posterior cingulate are harder to interpret. The role of posterior cingulate in semantic cognition is unclear, though it has been suggested that it may be involved in the interface between semantic knowledge and episodic memory (Binder et al., 2009). Stronger claims have been made about the function of angular gyrus. Binder et al. (2009) proposed that the angular gyrus is critically involved in semantic representation and that concrete regions activate this region strongly because they have more detailed semantic representations. It is therefore interesting that the activation profile of angular gyrus diverged strongly from that of the vATL, for which a similar representational function has been proposed. There were three findings that suggest the function of the angular gyrus is very different to that of vATL. First, the angular gyrus was not activated in the main contrast of semantics over numbers; in fact, the contrast in this region slightly favoured the numbers (see Fig. 3). This suggests that, in addition to any putative role in semantic processing, the angular gyrus is at least equally involved in the processing of numerical magnitudes. This was not the case for vATL. Second, angular gyrus showed a clear C > A activation pattern, while the activation in the vATL slightly favoured abstract words (though this effect varied elsewhere in the ATL region).

Finally, angular gyrus and vATL showed very different activation patterns with respect to rest, with angular gyrus significantly deactivated by the semantic task while vATL, along with other elements of the semantic network, were positively activated. This result is consistent with the status of angular gyrus as a key element of the default mode network. Binder et al. (2009, 1999) have proposed that much default mode activation can be attributed to ongoing semantic activity that occurs when participants “mind-wander” in the absence of a specific task. In other words, it is possible that participants were engaging in deeper semantic processing during rest/fixation than they are during the explicit semantic task and this could explain why the angular gyrus appeared to be deactivated in the semantic condition. However, this account does not explain why the angular gyrus was only putative semantic region to display deactivation, while other regions (ATL, IFG) showed strong positive activation. In summary, our results indicate that the role of angular gyrus is distinct from the representational and semantic control functions established for prefrontal and anterior temporal regions. Though its precise role is not clear as yet, we note that angular gyrus is positively activated by a range of non-semantic tasks, including numerical processing and episodic memory, suggesting that it may support more general attentional and working memory functions (Cabeza, Ciaramelli, & Moscovitch, 2012).

## Figures and Tables

**Table 1:** Example trials.

Condition	Cue	Judgement
Contextual cue	The road is closed. We must look for an alternative.	alternative substitute ambition discretion
	I ordered the roast dinner. It was served with asparagus.	asparagus broccoli christmas furniture

Irrelevant cue	He's very ignorant. He never takes a hint.	rate worth speed excuse
	We had sandwiches for lunch. They contained cucumber.	villain herring crook aluminium

Number baseline	Snakes can be dangerous. Some have deadly venom.	56 43 40 51
	My knee hurts. I have damaged the cartilage.	85 63 62 59

**Table 2:** Mean psycholinguistic properties of stimuli (range in parentheses).

Property	Abstract probes	Abstract choices	Concrete probes	Concrete choices
Log frequency	1.37 (.16–2.82)	1.36 (.19–2.92)	1.36 (.22–3.04)	1.34 (.05–2.73)
Length	6.9 (3–12)	7.1^a^ (3–13)	6.9 (3–12)	6.0 (3–12)
Imageability	295^a^ (220–429)	300^a^ (210–503)	581 (497–655)	572 (408–652)
Concreteness	304^a^ (217–421)	295^a^ (183–485)	570 (360–660)	558 (334–653)
Familiarity	476^a^ (195–606)	476^a^ (162–624)	530 (425–646)	522 (298–657)
Age of acquisition	518^a^ (282–664)	503^a^ (256–661)	322 (144–597)	330 (167–540)
Semantic diversity	1.85^a^ (1.13–2.29)	1.83^a^ (.59–2.29)	1.49 (.85–2.15)	1.52 (.57–2.11)

Log frequency = log-transformed lemma frequencies from the CELEX database (Baayen, Piepenbrock, & van Rijn, 1993). Length = number of letters. Imageability, concreteness, familiarity and age of acquisition ratings were obtained from the MRC database (Coltheart, 1981) and were supplemented with additional data from Bird, Franklin, and Howard (2001) and Stadthagen-Gonzalez and Davis (2006). Semantic diversity values were obtained from Hoffman, Lambon Ralph, et al. (2013).

a Indicates a significant difference between concrete and abstract words (*p* < .05).

**Table 3:** Behavioural data.

Concreteness	Cue	% Accuracy	Reaction time
Abstract	Contextual	94.8 (3.6)	1989 (271)
	Irrelevant	86.5 (5.6)	2323 (225)
Concrete	Contextual	98.1 (1.8)	1710 (247)
	Irrelevant	97.1 (2.9)	1870 (260)
Number baseline		95.1 (3.7)	2138 (258)

Standard deviations in parentheses.

**Table 4:** Activation peaks for synonyms minus numbers contrast.

Location	Extent (voxels)	*Z*	MNI co-ordinates		
			*x*	*y*	*z*
L inferior frontal gyrus	1437				
Pars triangularis		5.94	**−52**	**28**	**10**
Pars orbitalis		4.13	−48	26	−18
Pars orbitalis		4.12	−56	22	−6
L ventral temporal lobe	645				
Anterior fusiform & ITG		5.77	**−42**	**−14**	**−34**
Posterior fusiform		4.59	−44	−46	−28
Posterior fusiform		4.08	−42	−38	−20
L superior temporal lobe	1514				
Posterior STS		5.26	−46	−34	0
Posterior STS		4.92	−44	−44	4
STG/supramarginal gyrus		4.64	−60	−46	20
Anterior STS		4.58	**−54**	**6**	**−22**
L occipital lobe	799				
Lingual gyrus		5.98	−16	−94	−14
Lingual gyrus		5.01	−28	−86	−16
R lingual gyrus	209	5.04	16	−92	−8
R post–central gyrus	69	4.78	36	−24	54
L TPO junction	56	3.98	−38	−60	24
R cerebellum	147	3.89	30	−72	−38
			20	−76	−38
			20	−68	−32
L cerebellum	57	3.67	−20	−84	−46

Peaks in boldface were used for ROI analysis of condition effects. ITG = inferior temporal gyrus; STG = superior temporal gyrus; STS = superior temporal sulcus; TPO = temporoparietal occipital.

**Table 5:** Effects of concreteness and cue type in regions of interest.

ROI	Concreteness	Cue type	Concreteness × cue type
IFG	Abstract > concrete *F*(1,18) = 17.7, *p* = .001	Irrelevant > contextual *F*(1,18) = 6.70, *p* = .019	No interaction *F* < 1
sATL	Abstract > concrete *F*(1,18) = 34.5, *p* < .001	Contextual > irrelevant *F*(1,18) = 6.06, *p* = .024	No interaction *F* < 1
vATL	Abstract > concrete *F*(1,18) = 8.50, *p* = .009	No effect *F*(1,18) = 2.79, *p* > 0.1	No interaction *F* < 1

IFG = inferior frontal gyrus; sATL = superior anterior temporal lobe; vATL = ventral anterior temporal lobe.

**Table 6:** Activation peaks for whole-brain analysis of concreteness effects.

Location	Extent (voxels)	*Z*	MNI co-ords		
			*x*	*y*	*z*
*Abstract > Concrete*					
L inferior frontal gyrus	786				
Pars orbitalis		4.57	−48	32	−4
Pars triangularis		4.56	−56	26	2
Anterior insula		4.55	−28	26	−2
L anterior STS	299	4.83	−54	10	−18
L anterior ITG & fusiform	213	4.62	−42	−4	−42
		3.87	−44	−12	−34
L posterior STS	663	4.27	−50	−26	−4
		3.99	−48	−36	0
		3.93	−40	−44	10
L STG/supramarginal gyrus	100	3.95	−50	−46	20
		3.78	−58	−44	22
L lingual gyrus	59	4.55	−38	−86	−12
L lingual gyrus	256	4.48	−20	−90	−12
R inferior frontal gyrus	87	3.66	40	32	−6
R anterior STS	53	3.97	54	14	−20
R posterior MTG	223	4.62	48	−28	2
		4.38	36	−40	2
R lingual gyrus	332	4.47	32	−90	−10
R cerebellum	302	4.01	16	−74	−24
		3.83	26	−68	−40
		3.80	24	−70	−26
*Concrete > Abstract*					
L rostromedial frontal lobe	98	4.42	−8	60	8
L posterior fusiform/PHG	96	4.28	−26	−38	−18
		3.81	−26	−30	−22
L dorsal precuneus/cingulate	60	4.29	−12	−38	58
L angular gyrus	138	4.07	−46	−78	22
		3.59	−42	−74	34
R superior frontal gyrus	52	3.93	24	24	46
R angular gyrus	219	3.71	44	−68	32
		3.61	46	−62	38
		3.55	40	−56	24
Bilateral posterior cingulate/precuneus	458	4.32	12	−58	14
		3.92	−2	−58	22
		3.61	−12	−50	28

STS = superior temporal sulcus; ITG = inferior temporal gyrus; MTG = middle temporal gyrus; STG = superior temporal gyrus; PHG = parahippocampal gyrus.

## Footnotes

1 ATL is a general term which is used in slightly different ways by different researchers. In this study, we use the term ATL to refer to the entire anterior third of the temporal lobe. We use the more specific term ventral ATL (vATL) to refer to the anterior third of the fusiform and inferior temporal gyri and superior ATL (sATL) to refer to the anterior third of the superior temporal gyrus and superior temporal sulcus.
