# Supplementary material for: Differing contributions of inferior prefrontal and anterior temporal cortex to concrete and abstract conceptual knowledge
Source: Cortex. 2015 Feb;63:250–66. doi: 10.1016/j.cortex.2014.09.001 (PMC4317194; doi:10.1016/j.cortex.2014.09.001)
Supplement: Supplementary file 1 [file mmc1.docx]

Supplementary Materials

Latent semantic analysis of stimuli: We used latent semantic analysis ([Landauer and Dumais, 1997](#_ENREF_3)) to investigate the semantic relationships between our cues and decision words. This technique involves data reduction performed on a large corpus. It represents words in a high-dimensional semantic space, with the proximity of two words indicating the degree to which they are used in similar linguistic contexts. Similarity in contextual use is taken to indicate relatedness in meaning. LSA was performed on the British National Corpus, using the method described by Hoffman et al. ([2013](#_ENREF_1)). We used the cosine of the angle between the vectors representing two words as measure of their semantic relatedness. This allowed us to assess semantic relationships between probes and their semantically-related targets, as well as with their unrelated foils. To obtain a semantic representation of the cues, we averaged the vectors of all the individual words in the cue ([Landauer, 2001](#_ENREF_2)). We then compared these using the cosine method assess relatedness between the words in the decision and their cues, and to the mean cosine values for concrete and abstract trials are shown below (standard deviations in parentheses).

| Relationship | Abstract trials | Concrete trials |
| --- | --- | --- |
| Probe with target | .19* (.13) | .39 (.24) |
| Probe with foils | .02* (.08) | .00 (.07) |
| Contextual cue with probe | .23* (.16) | .30 (.20) |
| Contextual cue with target | .09* (.08) | .16 (.14) |
| Contextual cue with foils | .03* (.07) | .01 (.05) |
| Irrelevant cue with probe | .03* (.06) | .01 (.07) |
| Irrelevant cue with target | .02 (.06) | .01 (.05) |
| Irrelevant cue with foils | .03* (.07) | .01 (.05) |

* indicates a significant difference between concrete and abstract trials (*p* < 0.05).

References

Hoffman P, Lambon Ralph MA, and Rogers TT. Semantic diversity: A measure of semantic ambiguity based on variability in the contextual usage of words. *Behavior Research Methods,* 45(3): 718-730, 2013.

Landauer TK. Single representations of multiple meanings in latent semantic analysis. In Gorfein DS (Ed.) *On the consequences of meaning selection: Perspectives on resolving lexical ambiguity*. Washington, D. C.: APA Press, 2001: 217-232.

Landauer TK and Dumais ST. A solution to Plato's problem: The latent semantic analysis theory of acquisition, induction and representation of knowledge. *Psychological Review,* 104: 211-240, 1997.
